# Time for actions in lucid dreams: effects of task modality, length, and complexity

**DOI:** 10.3389/fpsyg.2013.01013

**Published:** 2014-01-16

**Authors:** Daniel Erlacher, Melanie Schädlich, Tadas Stumbrys, Michael Schredl

**Affiliations:** ^1^Institute of Sport Science, University of BernBern, Switzerland; ^2^Institute of Sports and Sports Sciences, Heidelberg UniversityHeidelberg, Germany; ^3^Medical Faculty Mannheim, Central Institute of Mental Health, Heidelberg UniversityMannheim, Germany

**Keywords:** lucid dreaming, time, motor activity, cognitive activity, REM sleep

## Abstract

The relationship between time in dreams and real time has intrigued scientists for centuries. The question if actions in dreams take the same time as in wakefulness can be tested by using lucid dreams where the dreamer is able to mark time intervals with prearranged eye movements that can be objectively identified in EOG recordings. Previous research showed an equivalence of time for counting in lucid dreams and in wakefulness (LaBerge, 1985; Erlacher and Schredl, 2004), but Erlacher and Schredl (2004) found that performing squats required about 40% more time in lucid dreams than in the waking state. To find out if the task modality, the task length, or the task complexity results in prolonged times in lucid dreams, an experiment with three different conditions was conducted. In the first condition, five proficient lucid dreamers spent one to three non-consecutive nights in the sleep laboratory. Participants counted to 10, 20, and 30 in wakefulness and in their lucid dreams. Lucidity and task intervals were time stamped with left-right-left-right eye movements. The same procedure was used for the second condition where eight lucid dreamers had to walk 10, 20, or 30 steps. In the third condition, eight lucid dreamers performed a gymnastics routine, which in the waking state lasted the same time as walking 10 steps. Again, we found that performing a motor task in a lucid dream requires more time than in wakefulness. Longer durations in the dream state were present for all three tasks, but significant differences were found only for the tasks with motor activity (walking and gymnastics). However, no difference was found for relative times (no disproportional time effects) and a more complex motor task did not result in more prolonged times. Longer durations in lucid dreams might be related to the lack of muscular feedback or slower neural processing during REM sleep. Future studies should explore factors that might be associated with prolonged durations.

## Introduction

The question of time in dreams is frequently debated in science, philosophy and recently also by Hollywood film makers. For instance, in the movie *Inception* (Nolan and Thomas, 2010), dream time runs much slower than real time, 5 min of real time equaling 1 h of dream time. The idea, which inspired Christopher Nolan, the director of Inception, that time is scaled down during dreams, can be traced back a century and a half to the work of the French scholar Alfred Maury (1861), who was convinced that dreams are created at the moment of waking up. He based this assumption on a subjectively long-lasting dream about the French Revolution, at the end of which the dreaming Maury was to be beheaded under the guillotine. When he was roughly awoken by a piece of his bed (la flèche de mon lit) which had fallen on his neck, Maury assumed that the whole dream had been created at that very moment, leading up to the guillotine scene.

Maury's dream explanation led to the so-called Goblot hypothesis. In 1896 the French logician Edmond Goblot (1896) proposed that remembered dreams occur during the process of awakening and that a difference exists, therefore, between the time experienced in a dream and the time which actually passes while the dream is taking place. Hall (1981) tried to find evidence to support the Goblot hypothesis by showing that stimuli of a sleeper's surrounding as well as internal stimuli, such as hunger, were represented in the dreams of his subject who had recorded his dreams for two years. While such correspondence was found to some extent, Hall admitted himself that this does not prove that these dreams are generated during awakening, as external and internal stimuli “… are or may be present while we are asleep or before we go to sleep” (Hall, 1981, p. 245). In this approach the assumptions concerning time in dreams were indirect implications of a hypothesis on the origin of dreams in general. The idea that dreams are instantaneous memory insertions experienced at the moment of awakening also plays a major role in philosophical debates, for example in Dennett's cassette-theory of dreaming (Dennett, 1976).

A few years after the discovery of rapid-eye movement (REM) sleep and its initial association with dreaming (Aserinsky and Kleitman, 1953), Dement and Kleitman (1957) explored more precisely the relationship between REM sleep and dream activity. In one of their experiments, they wanted to demonstrate the relation between the lengths of periods of rapid eye movements and the subjects' estimations of how long they had been dreaming. In their study, participants were awakened randomly, either 5 or 15 min after REM onset, and were then asked if they had dreamed 5 or 15 min. In 92 out of 111 awakenings (83%) the participants judged correctly. The authors also found a correlation between the elapsed amount of time and length of dream reports (*r* = 0.40 to *r* = 0.71). These results were replicated by other researchers (e.g., Glaubman and Lewin, 1977; Hobson and Stickgold, 1995) and nowadays it is a widely accepted hypothesis that subjectively experienced time in dreams corresponds with the actual time. Yet, a study conducted by Moiseeva (1975) found that in dreams with a complex and bizarre structure or in very emotional dreams, time can be perceived as flowing much faster, exceeding the absolute time span of a dream by 2–10, 25–50 or even 100 times.

While in regular dream studies, this correspondence can only be explored on a correlational basis and retrospectively, a completely different approach opens when conducting studies with lucid dreamers. A lucid dream is defined as a dream during which dreamers, while dreaming, are aware they are dreaming (LaBerge, 1985). Lucid dreams are considered to be mainly REM sleep phenomena (LaBerge, 1990). Lucid dreamers can consciously influence the dream content and are thus able to carry out prearranged tasks while dreaming (e.g., Fenwick et al., 1984; Erlacher and Schredl, 2008a, 2010). In order to mark events or actions in a lucid dream, lucid dreamers can produce a specific pattern of eye movements (e.g., left-right-left-right) that can be objectively identified on an electrooculogram (EOG) recording (cf. Erlacher et al., 2003). Lucid dreams are especially useful for studying time intervals in the dream state because the beginning and end of a certain action can be marked with eye signals while the sleep is recorded using standard polysomnography.

In general, lucid dream studies conducted in sleep laboratories demonstrated that a certain time is needed during the recorded REM period. However, only two studies explored time in lucid dreams explicitly. In a pilot study, LaBerge (1985) demonstrated that the time interval for counting from one to ten in a lucid dream is about the same compared to that of wakefulness. Erlacher and Schredl (2004) investigated the duration of a sequence of squats (deep knee bends) compared to what would have been necessary in wakefulness. Five participants performed the following task both in wakefulness and while dreaming lucidly: Counting five seconds, performing ten squats and counting five seconds again. By means of eye signals, the durations of each counting or squat sequence could be determined and compared to the duration of waking performances. While there was no significant difference between wakefulness and dream state for the counting intervals, participants required about 40% more time for performing squats in lucid dreams than in the waking state. This finding contradicts the results of prior studies which supported equivalence of dream time and physical time.

Different explanations can be used to explain why more time was required for performing squats in the dream state. Firstly, there might be a difference between the task modalities. For example, tasks that involve an activation of the body concept in the dream could require more time due to a more complex simulation of this body schema. Secondly, there might be a difference due to the task duration: In the study described above by Erlacher and Schredl (2004), the motor task (*M* = 17.84 s, *SD* = 6.8) lasted almost three times as long as the counting task (first counting: *M* = 6.26 s, *SD* = 1.7; second counting: *M* = 6.48 s, *SD* = 1.0), when measured in wakefulness. Therefore it might be possible that longer tasks generally lead to increased durations in the dream state. Further, if there is indeed a need for more complex simulation to take more time in the dreaming state, then more complex actions in the dream should also lead to longer durations.

In the present study we conducted further experiments to explore the effects of task modality (involving motor activity vs. not involving motor activity), length (intervals of 10, 20, or 30 s/steps), and complexity (simple motor task vs. complex motor task) on task durations in lucid dreams. The durations of three different tasks were compared in wakefulness and in lucid dreams: counting, walking and a gymnastic routine.

## Materials and methods

### Participants

Participants were recruited either from previous studies or by advertisement via different media about lucid dreaming, including a German web page (http://klartraum.de), or from lucid dream induction studies in which specific techniques were applied in order to induce lucidity (e.g., MILD, LaBerge, 1980). Table 1 depicts the participants who successfully finished one of the three experimental protocols (the walking and gymnastic tasks included not only lucid dreamers but also sports students who participated in a lucid dream induction study. The average lucid dream frequency in these groups was thus somewhat lower). Informed consent was obtained from the participants and participation was paid.

**Table 1:** **Participants characteristics**.

	***N* (gender)**	**Age (years)**		**Dream recall frequency frequency (dreams/week)**		**Lucid dream recall frequency (lucid dreams/month)**		**Frequent lucid dreamers**
		***M***	***SD***	***M***	***SD***	***M***	***SD***	
Counting^a^	5 (4 males, 1 female)	28.2	4.8	5.4	2.5	14.4	7.9	4
Walking^a^	8 (5 males, 3 females)	26.4	4.5	4.4	2.5	9.1	9.5	4
Gymnastic	8 (2 males, 6 females)	25.3	4.3	3.9	2.5	7.3	9.0	4

^a^All participants of the counting task also completed the walking task.

### Experimental conditions

The task descriptions for the three conditions:

#### Counting

**Figure 1:**
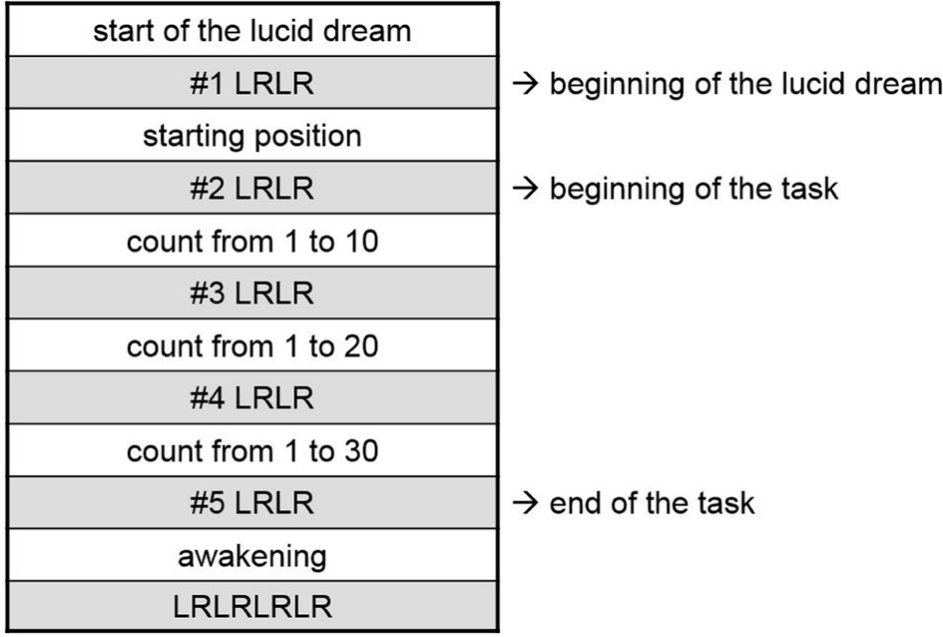
**Experimental protocol for the lucid dream task (counting)**.

For the counting task, participants had to count from 1 to 10, from 1 to 20, and from 1 to 30 at their own regular pace. During counting, participants were asked not to move (see Figure 1 as an example).

#### Walking

For the walking task, participants had to walk 10 steps, 20 steps, and 30 steps at their own regular pace.

#### Gymnastic routine

The gymnastic routine consisted of four consecutive elements starting in an upright position with feet together. Participants were instructed to count along while performing the elements (see Supplement 1):

Count 1, 2: Straight jump, landing with feet apart to the left and right, straight jump, putting feet together again

Count 3, 4: Straight jump, landing with feet apart to front and back, straight jump, putting feet together again

Count 5, 6, 7, 8: roll forward, standing up

Count 9, 10: Straight jump with half turn (180°)

For the counting and walking task, participants performed the task at their own regular pace. The gymnastic routine was developed to match the walking 10 steps condition regarding the task duration in wakefulness. The task was presented by the experimenter and the participants were asked to perform the task at the same speed and pace.

### Sleep recordings

In all studies, polysomnography was conducted to register the sleep stages. Sleep was recorded by means of the following standard procedures: electroencephalogram (EEG; C3 and C4 for counting and walking; F3, F4, C3, C4, O1, and O2 for gymnastic), EOG, submental electromyogram (EMG) and electrocardiogram (ECG). The data was recorded during the entire night (or during afternoon nap for one participant) by a standard recording device (XLTEK Trex Longtime EEG recorder or Schwarzer ComLab 32). Sleep stages for the counting and walking conditions were scored according to Rechtschaffen and Kales (1968) while those for the gymnastic condition were scored in accordance to the Manual of the American Academy of Sleep Medicine (2008).

### Procedure

The participants spent one to three non-consecutive nights in a sleep laboratory. One participant was recorded twice during an afternoon nap at about 3 pm.

Before sleep, participants received task instructions (see above) in written and oral forms. Afterwards, participants were instructed about left-right-left-right (LRLR) eye signals to mark task events in a lucid dream. The first signal was always to mark the onset of lucidity. In the counting and walking task participants had to mark the beginning of each task sequence as well as the end of the task (five signals for each successful dream). As an example, the exact protocol for the counting task is depicted in Figure 1. In the gymnastic routine, apart from the first signal for the onset of lucidity, only the beginning and the end of the task had to be marked (three signals for each successful dream).

**Figure 2:**
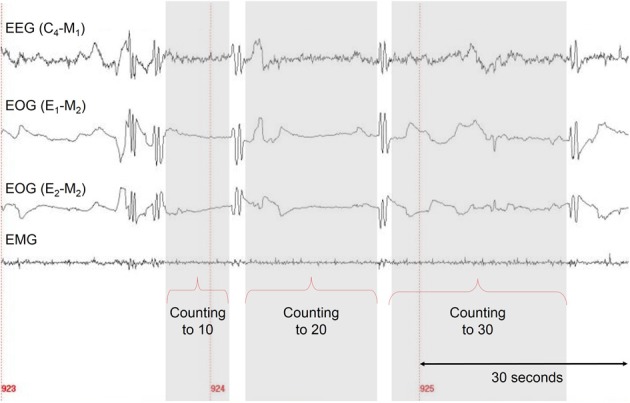
**A sample of one correctly signaled lucid dream for the counting task**. Five LRLR eye signals are depicted. The interval between two LRLR eye signals corresponds to the counting interval (gray area).

After the participants were familiar with the task and eye signaling, they carried out the task five times in wakefulness (including eye signals). In order to determine the duration of the task in wakefulness, in the counting and walking task the participants measured the times by themselves using a stopwatch—starting after the first eye signal and stopping with the onset of the second one. Because in the gymnastic routine it was not practical for the participants to handle the stopwatch, the experimenter started and stopped the times, according to a verbal signal from the participant, which was given immediately after and before the respective eye signal. For lucid dreams the time intervals were defined as the interval from the end of one LRLR eye signal to the beginning of the next LRLR and so on (see Figure 2).

During the night the experimenter monitored the recordings and woke participants up when recordings showed any of the following criteria: (1) A false awakening, i.e., the recording showing LRLRLRLR eye movements (signal for being awake, see below) but the EEG and EMG channel still showing characteristics for REM sleep. (2) Loss of lucidity, i.e., the recording showing five correct LRLR eye movements in the EOG channel, but no further eye signals occurring 30 s after the previous signal. These criteria were set in order to keep participants from sleeping on and forgetting specific parts of their lucid dreams (Erlacher and Schredl, 2008a). After accomplishing the task successfully in one lucid dream, the participants were to wake themselves up by the technique of focusing on a fixed spot in the lucid dream as described by Tholey (1983). In two cases the experimenter had to wake up participants after false awakening; in all other cases the participants woke up by themselves after finishing the lucid dream task (no cases of loss of lucidity).

The awakening had to be signaled by left-right-left-right-left-right-left-right eye movements (LRLRLRLR). After each lucid dream, participants wrote down a complete and precise dream report. Also they were asked whether they had been lucid and the task had been performed correctly by using a protocol which checked for each element of the task (e.g., eye signals). Any deviations from the protocol were highlighted (e.g., “only a single LR eye movement instead of a pair”) and evaluated to determine whether the data should be excluded. The complete set of dream reports used for data analysis can be found in Supplement 2.

### Excluded data

Out of *n* = 37 recorded lucid dreams *n* = 16 cases (counting: *n* = 2; walking: *n* = 4; gymnastic routine: *n* = 10) could not be used for the analysis. The criteria for inclusion of a data set were strict, in order to ensure that only lucid dreams conforming exactly to the protocol were used. A data set was excluded for one or more of the following reasons:

- One or more LR eye signals were not detectable in the recording (counting: *n* = 2; walking: *n* = 3; gymnastic routine: *n* = 5)
- An element of the task was skipped or the participant was unsure about having performed one or more of the elements (gymnastic routine: *n* = 1)
- The participant stated in the dream report that he or she had imagined the performance rather than carried it out “physically” (gymnastic routine: *n* = 2)
- The dream report showed that there was a delay between eye signal and task performance, e.g., one participant stated in the protocol that she had hesitated for a moment between the second eye signal and the start of the motor routine to recall the exact sequence of the task (gymnastic routine: *n* = 1)
- The dream content directly influenced the time of the task performance (walking: *n* = 1; gymnastic routine: *n* = 1).

To illustrate the last category, the two dream reports will be presented in detail (Original dream reports were in German, translations were done by the authors):

Dream example 1 Slow motion in the dream (gymnastic routine)

[longer dream sequence before] Then I did the LRLR and then I was here, the water was gone, but the floor was dark. I also felt that after this eye signal suddenly it was blurry again. I waited until it got better and then I walked around, wanting to find a brighter spot where I could see better and have more space. I went to a garden where it was bright and I thought, “Okay, I am doing the experiment now.” I gave a LRLR and I jumped and I felt immediately that jumping was very different compared to wakefulness. Just a different perception of the body, also slower. I continued and I did the forward roll—which lasted almost eternally. When I finished the task I gave a LRLR again”

The task duration was indeed 163% longer than in wakefulness (14.8 vs. 5.6 s).

Dream example 2: Running in the dream (walking)

[longer dream sequence before] We talked for about 5 min about the dream I had and that I often have nightmares. Suddenly, I was back at the party and saw the lights again but this time I realized that I was dreaming and did the LRLR. Afterwards I did the protocol but I was running instead of walking the steps. First 10, then 20 and then 30 steps. Finally I woke up”

The task duration was indeed significantly shorter than in wakefulness and therefore the data set was excluded for the statistical comparison of absolute times between wakefulness vs. lucid dream state (3.2). However, the data set is of special interest for the relative time and therefore it was included in the comparison of the relative timing analysis (3.1).

### Statistical analysis

Due to the small sample sizes, individual data are presented and analysis focuses mainly on a descriptive level. Furthermore, for the comparison of times between wakefulness and lucid dreaming, no predictions were made and, therefore, two-tailed statistical *t*-tests (dependent samples) as well as Wilcoxon tests were applied. For the comparison of task complexity, time differences between wakefulness and lucid dreaming for walking 10 steps and the gymnastic routine were calculated and two-tailed statistical *t*-test (independent samples) as well as Mann-Whitney-test applied. For all statistical tests a significance level of alpha = 0.05 was used. SPSS Statistics 20 software was used for the statistical analysis. For differences in times between wakefulness and lucid dreaming effect sizes d (Cohen, 1988) were calculated by the open-source software G^∗^Power V 3.1.3 (Faul et al., 2007). Cohen (1988) differentiated between small (*d* = 0.2), medium (*d* = 0.5), and large (*d* = 0.8) effect sizes.

## Results

### Absolute and relative times for counting, walking and gymnastics

Figure 2 shows a sample of a correctly signaled lucid dream for the counting task with five LRLR eye signals. The participant reported the following dream after awakening:

Dream example 3. Correctly signaled lucid dream (counting)

“I was awake and tried WILD [WILD stands for Wake-Initated Lucid Dream which is a technique to induce lucid dreams] which did not induce lucidity immediately. There was a long dream sequence where I had barbecue with some friend. Then I was in a basement with some cupboards and I played with some kids and adults. I knew that I was dreaming and I started to do the protocol: 1. LRLR for “I'm lucid,” 2. LRLR for counting from 1 to 10, 3. LRLR for counting from 1 to 20, 4. LRLR for counting from 1 to 30. After finishing the protocol I waited for a couple of seconds and the dream started to dissolve.”

**Figure 3:**
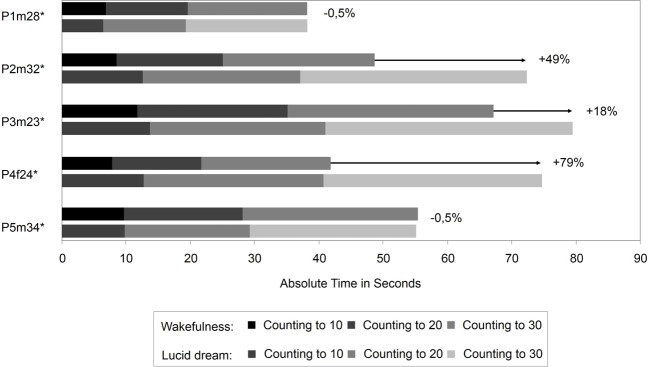
**Absolute durations for the counting task during wakefulness and lucid dreaming (Labels: e.g., P1m28 = Participant 1, male, 28 years**. ^∗^Participants of the counting task also completed the walking task).

**Figure 4:**
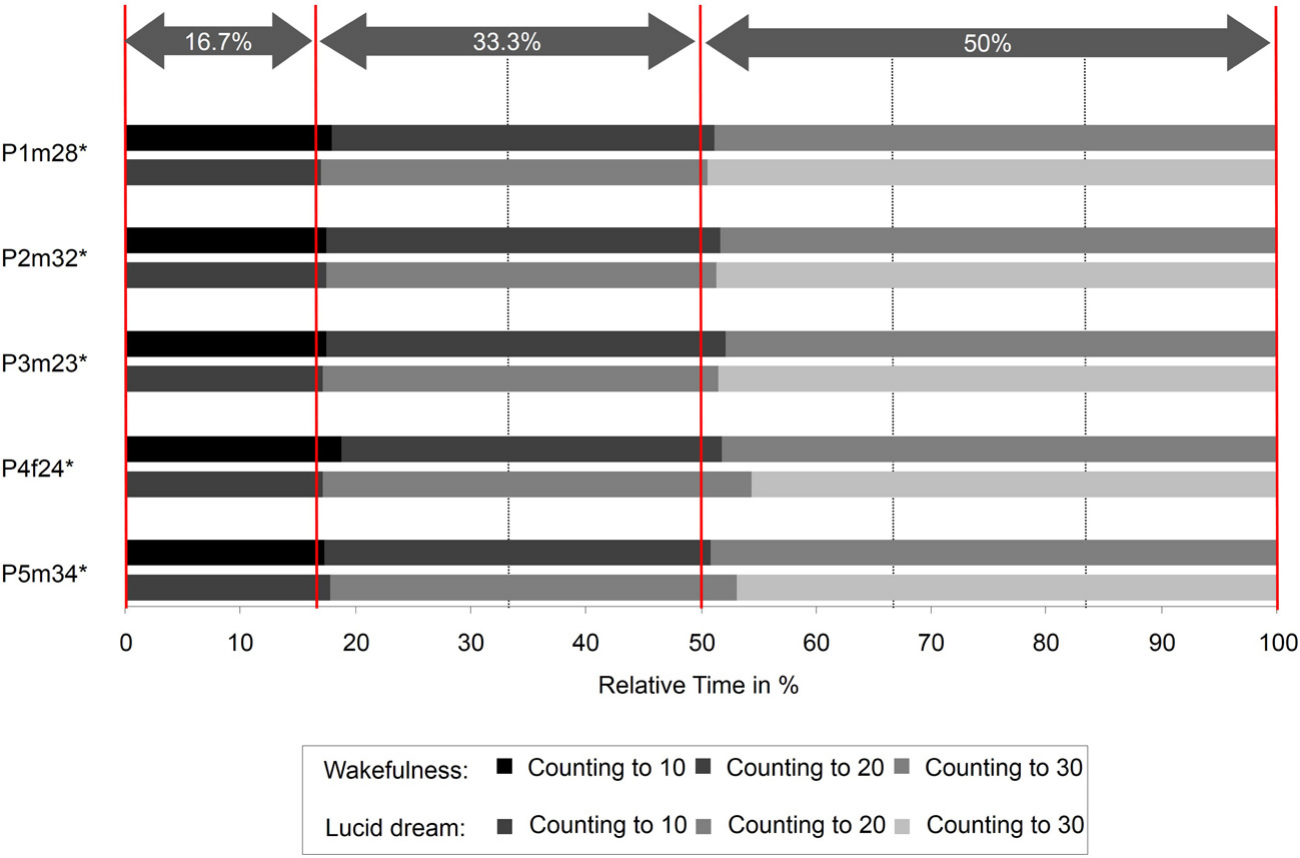
**Relative durations for the counting task during wakefulness and lucid dreaming (Labels: e.g., P1m28 = Participant 1, male, 28 years**. ^∗^Participants of the counting task also completed the walking task).

The interval between two LRLR eye signals corresponds to the counting interval (gray area). Figure 3 depicts the absolute times for the counting task during wakefulness and lucid dreaming. In three cases (P2m32, P3m23, P4f24) the absolute time was longer during lucid dreaming than in wakefulness. Figure 4 depicts the relative times for the counting task during wakefulness and lucid dreaming, e.g., the total time for the whole task equals 100%. Because the ratio for the three parts are 1/6 the expected relative time for counting from 1 to 10 is 16.7%, for counting from 1 to 20 is 33.3% and for counting from 1 to 30 is 50% (marked with the red lines in Figure 4). The differences between the expected percentage and the relative time structure of the counting task in wakefulness are *M* = 1.1% (*SD* = 0.6%), *M* = 0.5% (*SD* = 0.7%) and *M* = −1.5% (*SD* = 0.6%) and in lucid dreaming are *M* = 0.6% (*SD* = 0.3%), *M* = 1.6% (*SD* = 1.5%) and *M* = −2.2% (*SD* = 1.6%) (for counting to 10, 20, and 30, respectively).

**Figure 5:**
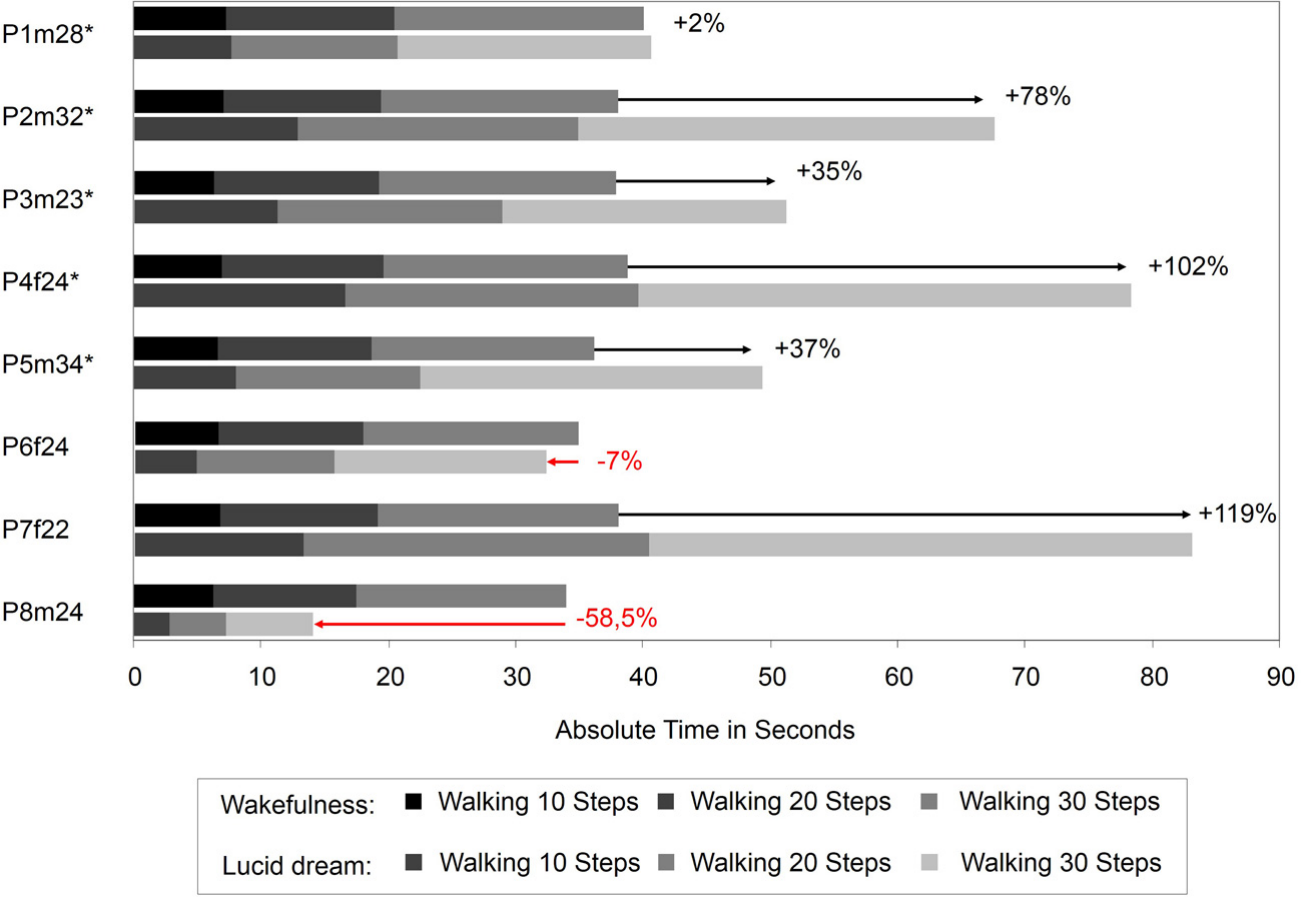
**Absolute durations for the counting task during wakefulness and lucid dreaming (Labels: e.g., P1m28 = Participant 1, male, 28 years**. ^∗^Participants of the counting task also completed the walking task).

**Figure 6:**
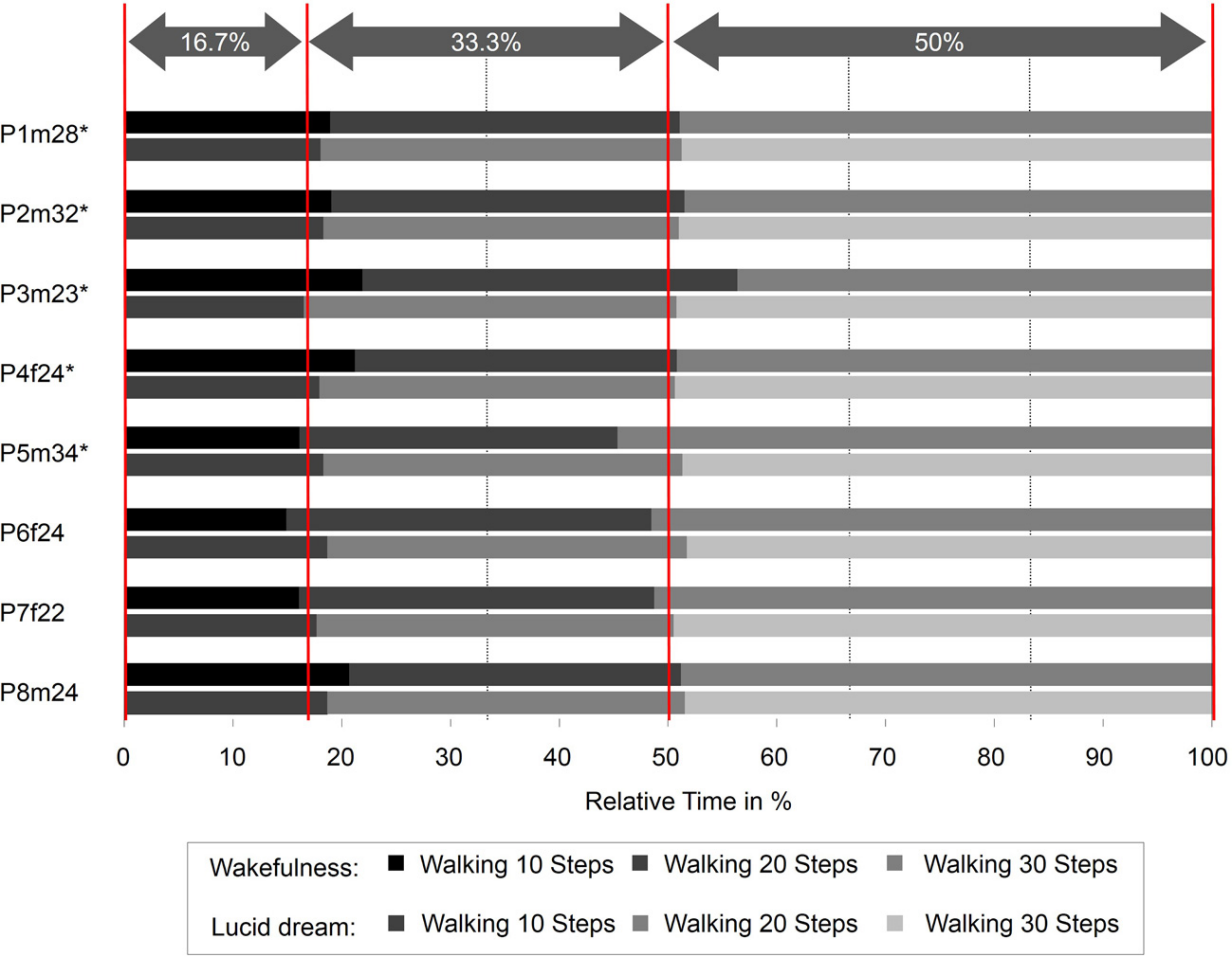
**Relative durations for the counting task during wakefulness and lucid dreaming (Labels: e.g., P1m28 = Participant 1, male, 28 years**. ^∗^Participants of the counting task also completed the walking task).

Figure 5 depicts the absolute times for the walking task during wakefulness and lucid dreaming. In five cases (P2m32, P3m23, P4f24, P5m34, P7f22) the absolute time was longer during lucid dreaming than in wakefulness. P8m24 exhibits significantly shorter time; however, the participant in this experiment experienced his first lucid dream and reported he was running instead of walking in the steps. Figure 6 depicts the relative times for the walking task during wakefulness and lucid dreaming, e.g., the total time for the whole task equals 100%. Again, the ratio for the three parts are 1/6 and the expected relative time for walking 10 steps is 16.7%, walking 20 steps is 33.3% and walking 30 steps is 50% (marked with the red lines in Figure 6). The differences between the expected percentage and the relative time structure of the walking task in wakefulness are *M* = 1.2% (*SD* = 0.8%), *M* = −0.2% (*SD* = 0.6%) and *M* = −1.0% (*SD* = 0.5%) and in lucid dreaming are *M* = 1.8% (*SD* = 2.7%), *M* = −1.5% (*SD* = 1.9%) and *M* = −0.3% (*SD* = 3.2%) (for walking 10, 20, and 30 steps, respectively).

**Figure 7:**
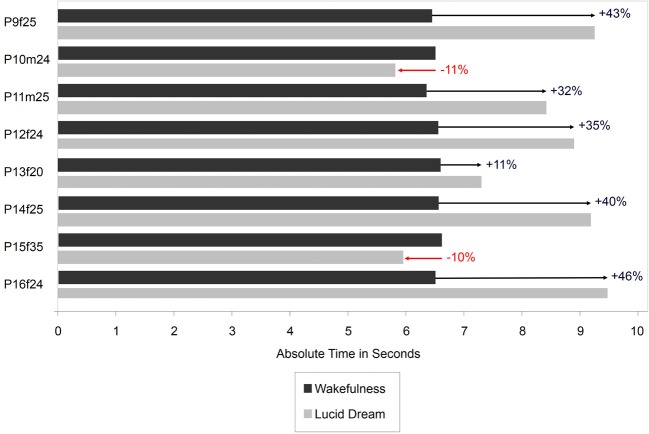
**Absolute durations of the gymnastic routing during wakefulness and lucid dreaming (Labels: e.g., P1f25 = Participant 1, female, 25 years)**.

Figure 7 depicts the absolute times for the gymnastic task during wakefulness and lucid dreaming. In six cases (P9f25, P11m25, P12f24, P13f20, P14f25, P16f24) the absolute time was longer during lucid dreaming than in wakefulness. In the other two cases (P10m24, P15f35) the duration of the gymnastic routine was slightly shorter in the lucid dream state than in wakefulness.

### Comparison of absolute times between wakefulness vs. lucid dream state

Table 2 summarizes the absolute times required for the counting, walking and the gymnastic task during wakefulness and lucid dreaming. For the counting and walking tasks, the total time is calculated by sum of counting to 10, 20, and 30 or walking 10, 20, and 30 steps. P8m24 was excluded for this statistical analysis because he was running instead of walking the 10, 20, and 30 steps. Statistically significant differences were found for the two tasks with motor activity, walking (*p* = 0.03) and gymnastics (*p* = 0.03) but not for the counting task (*p* = 0.10) (for statistical details see Table 2). In the lucid dream condition, the durations for counting were 27.2%, for walking 52.5% and for the gymnastic routine 23.2% longer than in wakefulness. The effect sizes for all three conditions were quite high (between 0.94 and 1.06), but for the counting task the statistical power was low (0.54).

**Table 2:** **Comparisons of times in wakefulness and lucid dreaming**.

	**Time in wakefulness (s)**		**Time in a lucid dream (s)**		**Difference**	**Wilcoxon-test**		***t*-test**			
	***M***	***SD***	***M***	***SD***	**%**	***Z***	***p***	***t***	***p***	**Effect size**	**Power**
Counting (*N* = 5)−Total	50.3	11.5	64.0	17.1	27.2	−1.2	0.23	2.10	0.10	0.94	0.54
Counting to 10	8.9	1.8	11.1	3.0	24.7						
Counting to 20	17.0	4.2	22.4	6.3	31.8						
Counting to 30	24.4	5.5	30.5	8.0	25.0						
Walking (*n* = 7^*^)−Total	37.7	1.7	57.5	19.2	52.5	−2.0	0.04	2.82	0.03	1.06	0.80
Walking 10 steps	6.7	0.3	10.6	4.0	58.2						
Walking 20 steps	12.5	0.6	18.3	6.0	46.4						
Walking 30 steps	18.5	0.9	28.6	9.8	54.6						
Gymnastic routine (*n* = 8)	6.6	0.1	8.1	1.5	23.2	−2.1	0.04	2.81	0.03	0.99	0.81

* P8m24 was excluded from this Table because he was running instead of walking the task.

### Comparison of walking 10 steps vs. gymnastic routine

**Figure 8:**
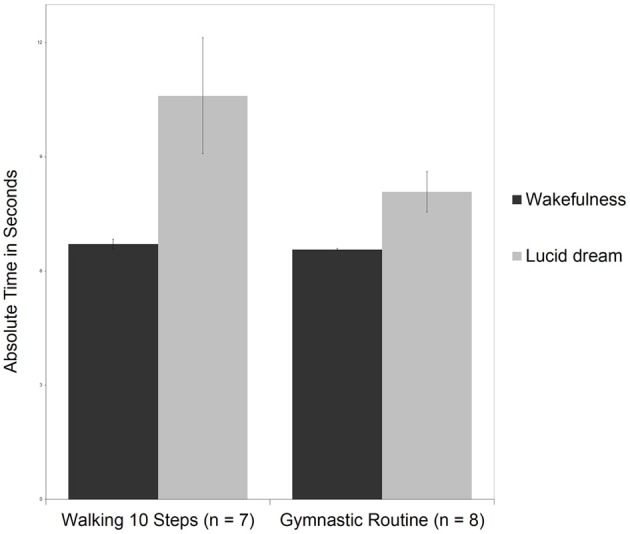
**Means and standard deviations for walking 10 steps and the gymnastic routine during wakefulness and lucid dreaming**.

Figure 8 depicts means and standard deviations for the walking 10 steps and the gymnastic routine during wakefulness and lucid dreaming. In wakefulness the gymnastic routine lasted *M* = 6.6 s (*SD* = 0.1) and therefore matched the time for walking 10 steps (*M* = 6.7 s, *SD* = 0.3). Comparing the two tasks with motor activity but different complexity, no statistically significant effects were found, *t*_(13)_ = 1.6, *p* = 0.14, *d* = 0.78, power = 0.42; Mann-Whitney-U: *Z* = 1.04, *p* = 0.30. Moreover, the more complex gymnastic routine required less time (8.1 s) than walking 10 steps (10.6 s) during lucid dreaming. Again, for this statistical analysis P8m24 was excluded because he was running instead of walking the 10, 20, and 30 steps.

## Discussion

In this study, longer durations were observed for all types of tasks in lucid dreams as compared to those when awake. The greatest increase in time was for walking (52.5%) while the lowest increase was for gymnastics (23.2%). The increase for counting was 27%, but did not reach statistical significance. The differences in time, however, were observed only for the absolute durations of the task, but not for the relative durations.

Before discussing the results, some limitations of the present study should be acknowledged. One of the biggest limitations is the small sample sizes. Small sample size is always related to statistical drawbacks because it is hard to determine if the data meet all prerequisites for parametrical testing (e.g., normality). In order to account for such statistical problems we, firstly, concentrated on presenting sufficient descriptive statistics and, secondly, ran additional non-parametric tests (Wilcoxon test). The obvious advantage of *t*-tests is that effect size (Cohens *d*) and test power can be calculated and therefore those results are presented in Table 2. Because in this study effect sizes are large (*d* > 0.8) and test power ranges from 0.5 to 0.8 the probability for type II error is high (as in the case of counting).

Increasing sample size in lucid dream studies is not easy because the enrolment of proficient participants is always complicated. In a representative survey by Schredl and Erlacher (2011) it was shown that about 50% of the population experienced at least one lucid dream, however only 1.2% have lucid dreams on a very frequent basis (e.g., several times a week) which is necessary for sleep laboratory studies. Further, in addition to becoming lucid, participants also need to remember the task, accomplish it, and produce unambiguous eye signals. A recent survey of lucid dreamers (Stumbrys et al., in press) showed that lucid dreamers are able to remember their waking intentions in lucid dreams in only about half of the occasions and only less than half of those remembered intentions can be successfully accomplished in lucid dreams (failures most often occur due to awakening or hindrances within the dream environment). This seems to be borne out by our own study: Recall that half of the data sets had to be excluded because dreamers failed to carry out the task.

Next, the sleep recordings for the present study were conducted over the period of several years and the electrode montage has slightly changed over the time. The first two conditions (counting and walking) were recorded in accordance with the guidelines by Rechtschaffen and Kales (1968), while the third condition (gymnastic) was recorded in accordance with the American Academy of Sleep Medicine (2008) guidelines.

It should also be mentioned that in the present study lucid dreams were used to explore a special feature of a motor routine and that the results and conclusion should not be generalized to “the dream state” as a matter of course. Dreams in general—referring to REM dreams—also include non-lucid dreams. An EEG study by Voss et al. (2009) indicated that there might be a difference between lucid and non-lucid REM sleep concerning frontal lobe activation. These findings are supported by Dresler et al. (2012) who demonstrated in an EEG/fMRI study that during the lucid dream state a network of different brain areas appear to be reactivated which are normally deactivated during REM sleep (including prefrontal, occipito-temporal cortices, precuneus, cuneus, parietal lobules). These studies do not indicate differences between lucid and non-lucid dreams concerning motor activity *per se*. However, we cannot simply exclude such a difference a priori. Future studies using EEG/fMRI recordings should also investigate motor activation during non-lucid dreams, based upon the correlation of activation patterns and reported motor activity.

It is also worth mentioning that in our study the counting and walking task was performed at the participants' own regular pace, e.g., counting to 10 did not match 10 s of physical clock time (see also Table 2). LaBerge (1985) for example explicitly trained his participants to estimate a specific interval of time as accurately as possible, namely 10 s by counting “One thousand and one, one thousand and two, … one thousand and ten” at a rate attempting to match 10 s of physical clock time. In our study for the counting and walking condition, we did not intend to match the lucid time durations exactly to physical clock time (e.g., 10 s). This allows participants to do the task at their own pace and has the advantage that they don't have to pay attention to this additional demand of concentrating to match a certain time interval. However, for the gymnastic routine the participants were trained to match the walking 10 steps condition regarding the task duration in wakefulness.

### Effects of task modality

Two different task modalities were used in the present study: those involving motor activity (walking and gymnastic conditions) and those not involving motor activity (counting condition). While increased durations in lucid dreams were observed for both modalities, only tasks with motor activity resulted in significant increases in time (with the caution of possible type II error for counting). These findings are in accordance with Erlacher and Schredl (2004) who also demonstrated that a task involving motor activity (performing squats) yielded an increased duration in lucid dreams. In contrast, tasks which did not involve motor activity (counting) led to negligible differences between wakefulness and lucid dreaming (3.5 and 9.6%). Also no differences were found in study by LaBerge (1985). However, in the present study the difference for counting was considerably higher (27.2%) and it is possible that only the small sample size did not allow it to reach statistical significance. Thus, while prolonged times are quite consistent across the range of different tasks involving motor activity (walking, gymnastics, performing squats), the findings regarding tasks without motor activity (counting) are still inconclusive.

It is important to note that all our conditions actually involved counting. Thus it is possible that the counting itself had an influence on the duration of the motor tasks. Therefore motor tasks which do not involve counting should be investigated in future studies in order to find out if the prolonged durations can still be found and if the extent of a probable increase is smaller or higher than when counting is involved.

Taking a closer look, there was also motor activity in the counting condition because participants were asked to count aloud. Even though the motor activation of the muscles involved during counting seems negligible in contrast to the gross motor activation during walking or the gymnastic routine, future studies should explore the difference for counting aloud and silent.

### Effects of task length

In two conditions (counting and walking), in addition to the absolute task time, also interim task times (after counting to 10 and to 20; and after walking 10 and 20 steps) have been measured. The analysis showed that relative times for both conditions did not differ between wakefulness and the lucid dream state. This was also true for one participant who accidentally ran the 10, 20, and 30 steps in his dream. Therefore it appears that extended durations in lucid dreams are not dependent on the task length or, in other words, there is not a disproportional time effect when accomplishing longer tasks.

It is worth mentioning that we did not randomize the order of lengths (e.g., P1: 10, 20, 30; P2: 30, 20, 10; etc.). This might confound the results with respect to order effects, however, one might speculate that possible order effects should have distorted the relative times in a systematic proportional way, but this was not the case.

### Effects of task complexity

Two different tasks with motor activity were included in the present study: a simple motor task (walking) and a complex motor task (gymnastic routine). While both motor tasks resulted in increased durations in lucid dreams, greater complexity of the task was not associated with greater increases in time. In fact, the trend was even in the opposite direction: Highest increases were observed for the most simple task, walking (52.5%), followed by somewhat more complex task from a previous study, performing squats (39.9%; Erlacher and Schredl, 2004), and finishing with the lowest increases for the most complex task, gymnastic routine (23.3%). While it is not clear if these differences just occurred by chance or there is indeed some inverse relationship between the task complexity and prolonged durations in lucid dreams, from the present data we conclude that more complex actions do not lead to longer durations.

However, it is important to acknowledge, that it is nearly impossible to provide an exact definition of “complexity” (Wulf and Shea, 2002) and the concept has been used in various ways. For example, Guillot and Collet (2005) use this notion in the sense of highly automatic movements (simple) in comparison to cyclical closed movements (complex). The gymnastic routine task, which has been employed in the present study, can be termed complex in several ways: it consisted of a sequence of different elements and was therefore a discrete as opposed to a continuous (walking) motor task. Also the various elements required higher levels of motor coordination and balance. It is still to be investigated whether and to what extent motor tasks which are complex in other ways than the gymnastic routine (e.g., regarding attention, task difficulty) affect dream state durations.

### Explaining extended durations

Since the difference in duration between wakefulness and the dream state was observed only for the tasks which involved motor activity, it is worth taking a look into studies which investigated the durations of motor tasks which were mentally simulated by participants while awake. Both in mental simulations and in the dream state motor activity is performed only in one's mind, without moving the physical body. Some mental simulation studies indeed found prolonged durations for mental simulations of walking tasks (Decety et al., 1989; Decety and Jeannerod, 1995) as well as in golf, swimming and weight lifting (for overview see Guillot and Collet, 2005). The difficulty of task, perceived force and skill complexity seem to be time-enhancing factors (Guillot and Collet, 2005). However, the findings from mental simulation studies are ambiguous: Some authors report equivalence of time (e.g., Munzert, 2002), others found shortened durations (Calmels and Fournier, 2001).

One possible explanation from mental stimulation studies for the prolonged durations might be centrally encoded force (Jeannerod, 1994). In the experiment by Decety et al. (1989) the participants who mentally simulated a walking task with an actual 25-kg weight on their back had increased mental simulation durations by about 30%. Jeannerod (1994) suggests that somehow the programmed increased level of force—as a reaction to the actual weight perceived—could not be used to overcome physical resistance and was thus misread by participants as a longer duration. Physically perceived force thus led to the program “increased effort required.” In dreams the perceived force, in the sense of gravity or resistance, might not correspond to the ordinary gravity force in wakefulness, because no real gravity force exists in the dream simulation and muscular feedback is lacking due to REM sleep atonia. Therefore the movements may also be programmed with “increased effort” to compensate for the lack of muscular feedback.

Another possible explanation might be related to neural specifics of REM sleep. Louie and Wilson (2001) found that when rats were trained in a behavioral task their hippocampal activity during the task in wakefulness was replayed in REM sleep but with a somewhat different temporal scaling factor. Most scaling factors were bigger than 1.0 (i.e., there was a slower corresponding activity during REM sleep) and the average was 1.4 ± 0.6. This average duration increase by 40% in REM sleep are in line with our findings on increased duration of motor tasks in lucid dreams (gymnastic: 23.3%; squats: 39.9%; walking: 52.5%). However, it is not clear if the observed replayed neural patterns are indeed linked to (dreamed) motor activity or if they rather represent learning procedures regarding temporal-spatial orientation. The task for the rats involved motor activity and therefore it is possible that the observed neural activity during REM sleep was connected to motor learning, although it is impossible to say if the rats actually dreamed of accomplishing the task. Louie and Wilson (2001) also found that the theta EEG rhythm during REM sleep was about 1.2 times slower compared to the practice in wakefulness and therefore provides two possible explanations. Firstly, this might reflect a globally slower neural processing during sleep due to lower brain temperature. Further, the theta rhythm itself might serve as a pacing mechanism to coordinate interactions during information processing across multiple brain regions.

Finally, it is important to underline that in each condition two participants also produced quite similar time or even slightly shorter times compared to wakefulness. Unfortunately, from our data it is not possible to conclude why those participants performed differently. For example, P1m28 was a highly frequent lucid dreamer and he showed very exact times in his lucid dreams. On the other side, P6f24, who also showed quite exact yet slightly shorter time in the walking condition during her lucid dream, was a very infrequent lucid dreamer.

### Implications for sports science

The relative timing of motor skills plays an important role in motor control theories. Schmidt (1975), for example, proposed in the motor schema theory that the relative time (e.g., the temporal structure of a motor skill) is an invariant component of a so-called generalized motor program and that parameters could scale this structure proportionately in time. For example, throwing a ball can be done fast or slow, however, the relative timing of the involved force impulse need to be proportional in order to speak of the same motor skill. If the relative time structure is not rigidly structured within a certain motor skill then this action is just something else but not the motor skill at hand (e.g., throwing a ball is no longer throwing but something else). The present findings of this study demonstrate that despite the longer absolute durations for tasks involving motor activity, the relative durations remain the same. This finding has important implications for lucid dream applications, such as using lucid dreams for motor skill practice: Athletes practicing long movement sequences seem to practice the same movement sequences as in wakefulness because the temporal structure is still given in their lucid dreams. With respect to relative time issues, it seems that lucid dreaming can be successfully applied for motor skill learning in sports (cf. Erlacher, 2007).

Practice in lucid dreams is similar to mental rehearsal in wakefulness: Movements are rehearsed with an imagined body on a cognitive level. Mental rehearsal is a well-established and widely used technique in sports science and practice. Meta-analyses (Feltz and Landers, 1983; Driskell et al., 1994) demonstrated that it has a positive and significant effect on performance. The evidence suggests that imagined and executed actions to some extent seem to share the same central neural structures. Decety (1996) presented three lines of evidence in support of this correspondence hypothesis: measurement of central nervous activity, autonomic responses, and mental chronometry. Similar correspondence can be demonstrated between dreamed actions in REM sleep and executed actions in wakefulness (Fenwick et al., 1984; LaBerge, 1990; Erlacher and Schredl, 2008b). The present study provides further evidence about the correspondence of mental chronometry (albeit with some scaling factor).

Previous studies with lucid dreamers demonstrated that complex sports skills, such as skiing or gymnastics, can indeed be successfully practiced in lucid dreams (Tholey, 1981). Also in this study, the participants were able to memorize a gymnastic routine and to recall and perform it within a lucid dream. It seems that athletes indeed are able to perform their sports in lucid dreams (Erlacher et al., 2011–2012) and that practice in lucid dreams can increase performance in wakefulness (Erlacher and Schredl, 2010).

### Future directions

The present findings should be replicated in future studies by using bigger sample sizes. It might be possible that not only experienced lucid dreamers can be involved, but also novices, supported by a lucid dream induction technique. In the third condition of the present study, some participants were not experienced lucid dreamers but sport students who took part in a lucid dream induction study. Nevertheless some of them were able to have their first lucid dream and successfully accomplished the requested task in it. A plethora of different methods have been suggested for lucid dream induction and some of them do look promising (see Stumbrys et al., 2012).

Future studies should explore the discrepancies found in the counting condition, as well a possible negative relation between the task complexity and prolonged times, i.e., that more simple motor tasks for some reason lead to longer durations. Further, measures of perceived effort (e.g., Borg, 1982) could be included to explore the relationship between prolonged durations and perceived effort when accomplishing a motor task. Also it might be worth investigating other possible influencing factors that were found to have an effect on durations in mental simulations, e.g., the level of expertise and task familiarity (Guillot and Collet, 2005). Concerning the features of the tasks used in our own studies, it might also be worth exploring possible differences between continuous (walking, squats) and discrete (gymnastic routine) motor tasks.

One of the difficulties with chronometric lucid dream research is that it mainly relies on subjective time perception. Therefore it would be interesting to approach this problem with another way of measuring the durations of dreamed actions by incorporating physical time intervals into lucid dreams with external auditory signals. In a recent study Strelen (2006) showed that in a lucid dream the dreamer can hear and distinguish an externally provided acoustic stimulus. These audio cues could serve as a start and stop signal of an interval, during which lucid dreamers, for example, could count numbers or count their steps while walking. The problem of subjectivity within dreams of course could not be avoided, however, this would allow another comparison of physical clock time with subjective time experience in lucid dreams.

## Conclusion

In summary, the present study confirms the findings of Erlacher and Schredl (2004) that motor actions lead to prolonged durations in lucid dreams. The findings for the durations of cognitive actions (without motor activity) are as yet inconclusive. The relative time structure of motor tasks that last longer in the dream state than in wakefulness do not result in disproportional task durations in the dream state. Lucid dreams, therefore, can be successfully applied for motor skill practice in sports, music and other areas. Prolonged durations might be related to the lack of muscular feedback or slower neural processing during REM sleep. Future studies should explore factors that might be associated with prolonged durations (e.g., level of perceived effort, continuous vs. discrete tasks, motor task with counting vs. without counting) and try to incorporate physical time intervals within the dream by external auditory signals (e.g., implementing audio cues as start and stop signals).

## Conflict of interest statement

The authors declare that the research was conducted in the absence of any commercial or financial relationships that could be construed as a potential conflict of interest.
