# Supplementary material for: Time for actions in lucid dreams: effects of task modality, length, and complexity
Source: Front Psychol. 2014 Jan 16;4:1013. doi: 10.3389/fpsyg.2013.01013 (PMC3893623; doi:10.3389/fpsyg.2013.01013)
Supplement: Supplementary file 1 [file DataSheet1.DOCX]

Supplement A. The gymnastic routine with four consecutive elements.

| Starting position: upright standing | **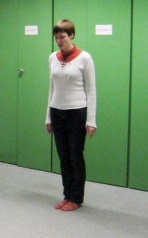** |
| --- | --- |
| **1. Jump:** Some kind of a straddle vault (like what the legs do when performing a jumping jack) without moving the arms: Feet apart and together again  **1… 2…** | **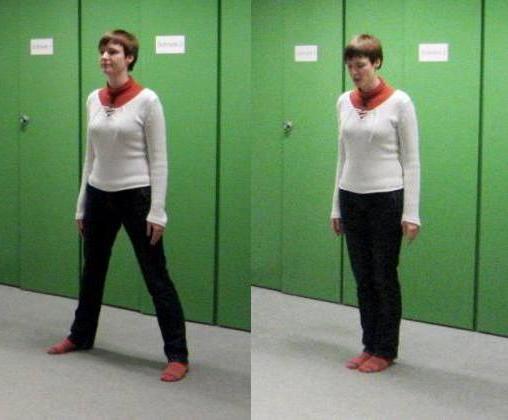** |
| **2. Jump:** Some kind of a scissor jump without moving the arms: one foot to the front, the other foot to the back and together again  **3… 4…** | **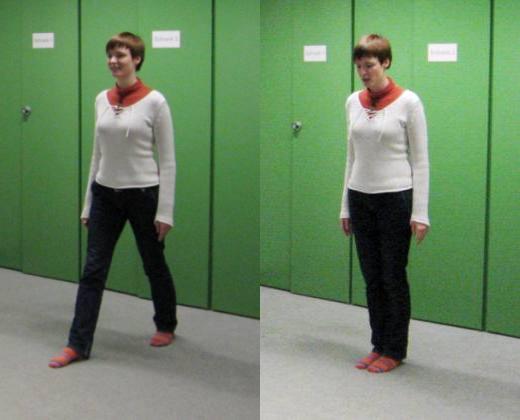** |
| **Roll:** Roll forward from standing position and back to standing position  **5… 6… 7… 8** | 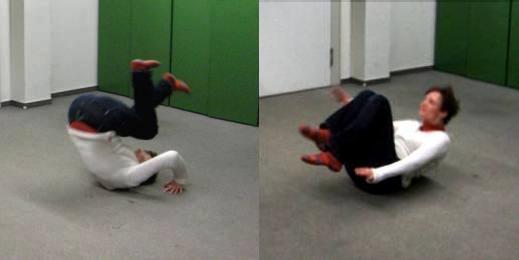 |
| **3. Jump:** Straight jump with half turn (180°), without moving the arms  **9… 10…** | 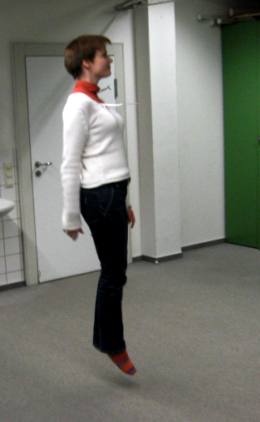 |
