# Supplementary material for: Time for actions in lucid dreams: effects of task modality, length, and complexity
Source: Front Psychol. 2014 Jan 16;4:1013. doi: 10.3389/fpsyg.2013.01013 (PMC3893623; doi:10.3389/fpsyg.2013.01013)
Supplement: Supplementary file 2 [file DataSheet2.DOCX]

**Supplement B – dream reports (**contains only those dream reports that were included in the analysis)

**Dream reports experiment 1 – counting**

**P1m28 (dream example 3 in the manuscript)**

I was awake and tried WILD [Wake-Initated Lucid Dream] which did not induce lucidity immediately. There was a long dream sequence where I had a barbecue with some friend. Then I was in a basement with some cupboards and I played with some kids and adults. I knew that I was dreaming and I started to do the protocol: the first LRLR for „I’m lucid“, the second LRLR for counting from 1 to 10, the third LRLR for counting from 1 to 20, the fourth LRLR for counting from 1 to 30 and the final LRLR for the end of the task. After finishing the protocol I waited for a couple of seconds and the dream started to dissolve.

**P2m32**

[longer dream sequence before] Now I decided to do the task. I did the whole counting task: Starting with the first LRLR, then counting to 10, then the second LRLR and counting to 20, then the third LRLR and counting to 30, and finally the fourth LRLR. I wondered if I should do the walking task, but decided to skip it and woke myself up.

**P3m23**

I felt that I was falling asleep and my body became lighter. I waited. Then I did a pinch test and I knew that I was dreaming. I was afraid that I 'm wired but I said to myself that this is a dream. I saw the experimenter and tried hard to wave my arms and legs to give a sound which I hoped that my sleeping body would also make. I saw that the experimenter noticed it and started to laugh. Then I concentrated on the task. I did a LRLR and counted from 1 to 10. I did another LRLR and counted from 1 to 20. I noticed that the dream became somewhat unstable and rubbed my hands [while counting - as explained by the participant afterwards]. I did another LRLR and counted from 1 to 30 and did a final LRLR. I woke up, however it was a false awakening. I talked to the experimenter. Finally I woke up entirely.

**P4f24**

I was in an apartment and my friend was there. Then I remembered that this cannot be true and I became lucid. I gave the LRLR and began to count from 1 to 10, then 1 to 20 and finally 1 to 30. Every time I gave also the LRLR. Then my lucidity disappeared and I woke up in a car. Here I read an SMS from my friend who wrote that he was at a TV show. Because of that, I realized again that I must be dreaming. So I went out of the car and began to do the task again. Here, I met a woman who suddenly started to hit me with her handbag. I could not mentally control the person and woke up.

**P5m34**

I was lying in bed, but I wanted to say something and stood up. I saw the cleaning woman in the diagnostic room and I realized immediately that this is a dream. I gave a LRLR signal and started with the lucid dream task: LRLR - Counting to 10 - LRLR - Counting to 20 - LRLR - Counting to 30 - LRLR. I tried to wake up, and had a short false awakening, before I finally woke up.

**Dream reports experiment 2 – walking**

**P1m28**

I induced a lucid dream by WILD and found myself in a dream. I did not want to waste time and started to do the protocol: the first LRLR for „I’m lucid“, the second LRLR for walking 10 steps, the third LRLR for walking 20 steps, the fourth LRLR for walking 30 steps. After finishing the protocol I woke myself up.”

**P2m32**

[longer dream sequence before] I was lucid when I remembered to do the task. I was just in a wide open field and made the first LRLR, then I walked 10 steps, then the second LRLR and I walked 20 steps, then the third LRLR and I walked 30 steps, and finally the fourth LRLR. The dream felt very stable. I would have certainly flown around if the experiment had not said that one should wake up immediately after the task itself, so that the memory of the task is still fresh. So I woke myself up.

**P3m23**

[longer dream sequence before] I was in the sleep laboratory and I became suspicious. I did a pinch test and felt no pain: I knew that I was dreaming. I made a LRLR for lucidity. I took the starting position and made a LRLR and walked 10 steps, LRLR, walked 20 steps, LRLR, walked 30 steps from the room into the hallway, LRLR. The dream was very stable and walking was not a problem at all. I woke myself up.

**P4f24**

I was going through my old college apartment where my furniture was still there. I suddenly realized that I was dreaming because the apartment should be empty. To do the task I took the fastest way out of the house and went through the wall of the living room. I ended up on a long, paved road without traffic. Here I went straight for the 10, 20 and 30 steps. In between, I stopped shortly and made the LRLR, as practiced in the evening. Then a man came along who tried to attack me. I bit in his hands and told him that I have to concentrate. Then I woke up.

**P5m34**

I tried to do the task once before, but I could not finish it, because the dream had dissolved. I did lots of LR eye movements and stabilized the dream by touching the wall. I was back in bed but still dreaming. I gave a LRLR for a new beginning and got up, then LRLR, 10 steps LRLR, 20 steps LRLR, 30 steps. The dream faded again and I had a false awakening. After about 10 seconds I woke up properly.

**P6f24**

[longer dream sequence before] Then I am suddenly in the sleep laboratory and I immediately realize that I am dreaming. I do the LRLR to signal that I'm lucid. Then again LRLR and begin to walking task: 10 steps, LRLR, 20 steps, LRLR, 30 steps, LRLR. The experimenter is in the same room and says "Too bad, now I've missed it." I woke up.

**P7f22**

I've lived with some people in a house. I went with two of them down into the garden. However, there was no seat for me and I had to get some coffee. In the garden everything was already decorated for Christmas, even though it was still summer and I knew that I was dreaming. In the hallway I did the steps and did the LRLRs.

**P8m24 (dream example 2 in the manuscript)**

[longer dream sequence before] We talked for about 5 minutes about the dream I had and that I often have nightmares. Suddenly, I was back at the party and saw the lights again but this time I realized that I was dreaming and did the LRLR. Afterwards I did the protocol but I was running instead of walking the steps. First 10 steps, then 20 steps and then 30 steps. Finally I woke up.

**Dream reports experiment 3 – Gymnastic Routine**

**P9f25***

I sat at a table at home with my father and my grandparents and my father started talking. At that point I heard the cue for the first time. I tried to focus on the cue, but he just kept on talking. Then my sister came and said that I had just had a lucid dream and my father affirmed: “Yes, she just had a lucid dream”. Then I did that reality check with the nose and it worked, I then gave the first eye signal and walked to the mat, there I did the second eye signal and then “One, two, three and so on…” I did the task. Then I did the eye signal again and then I stared at a fixed point, but at that point you [the experimenter] already woke me up.

**P10m24***

I was sleeping and I heard the cue. Then I heard it again and did the reality check. Then I did the eye signal, the task and again the eye signal and then I thought you [the experimenter] had told me it wasn’t clear enough and so I tried again. So I gave another eye signal and went to a place where the mat is, then eye signal again and then the motor task.

**P11m25***

I woke up in here, but in an open room and then an assistant came in and started to clean something with a wet cloth. He said: “We are done a bit earlier” and that he still needs to do some things, like measuring my weight and then he went to get something like a small medical suitcase where I could put the cables in. I then heard the cue and did the reality check and noticed that I could breathe in. I then did the eye signal and the gymnastic task. Then you woke me up [for analysis the second time was used].

**P12f24**

[longer dream sequence before] You [the experimenter] came in and you spooked me and you made “Wuuuuuah!” all the time. I mean, you were not a monster, but you somehow really annoyed me. Then I thought: “That can’t be true! This must be a dream!”, because you would not just walk in here, you would wake me via the microphone. Then I gave the eye signal, waited for a moment and prepared and stepped in front of the mat. It was here in this room, in front of the mat. Then I gave the eye signal again and performed the task and again the eye signal. And then that other girl came to wire me up again. That was the last thing before I woke up.

**P13f20***

I heard the cue again and then I did the task straight away. I thought the first signal was quite slow, however, the second [LRLR] should be okay. Then I did the task and completed with the last eye signal.

**P14f25**

I was talking to my family and in the middle of our chat I realized that I was dreaming. Then I was a bit scared to wake up. I rubbed my hands together and when I was in the dream again, I did the task. First I did the first eye signal, the one that says I am dreaming and then some time passed, before I did the second eye signal. Then I did the task and I focused on the eye signals. The forward roll felt funny, somehow rounder than in the waking state. I was not sure if it was here in this room and there was no mat on the floor. I had the impression that I was rather fast, because I was excited and I did not want to wake up.

**P15f35**

[longer dream sequence before] I was in a class room and there was a can of walnut milk and I wanted to get some and when I took something, everything was already on the floor and I thought: “No, that can’t be” and then I did the task twice, but the second time it was better, because for the second time I was outside on the grass and the first time it was inside and rather hectical. Then you woke me up [for analysis the second time was used].

**P16f24**

I was hitchhiking with trucks. Then we went along mountain streets, downhill and it was quite risky. And then I was shaving. At some point I thought: Okay, Now, I’ll do the task again. And then I did it [in a living room - as explained by the participant afterwards] and I think this time it went really well. And before and after the task I did the eye signals.

* In experiment 3 an acoustic cue was sometimes used to induce lucid dreams.
